# PRISMA-trAIce: A Name Without Endorsement

**DOI:** 10.2196/104210

**Published:** 2026-08-18

**Authors:** David Moher, Matthew Page, Joanne McKenzie, Yemisi Takwoingi, Evan Mayo-Wilson

**Affiliations:** 1 Centre for Journalology, Methodological and Implementation Research Program, Ottawa Hospital Research Institute, 501 Smyth Road, L1255, Box/C.P. 711, Ottawa, ON, K1H 8L6, Canada, 1 613-737-8899 ext 73812; 2 Methods in Evidence Synthesis Unit, School of Public Health and Preventive Medicine, Monash University, Melbourne, Victoria, Australia; 3 Department of Applied Health Sciences, College of Medicine and Health, University of Birmingham, Birmingham, England, United Kingdom; 4 Department of Epidemiology, UNC Gillings School of Global Public Health, University of North Carolina at Chapel Hill, Chapel Hill, NC, United States

**Keywords:** systematic literature review, reporting guideline, artificial intelligence, large language models, LLMs, PRISMA, transparency, evidence synthesis

We recently read the article titled “Transparent Reporting of AI in Systematic Literature Reviews: Development of the PRISMA-trAIce Checklist” by Holst and colleagues [1] in *JMIR AI*. We are writing in our capacity as members of the PRISMA Executive, which oversees and supports the development of the main PRISMA (Preferred Reporting Items for Systematic reviews and Meta-Analyses) statement (PRISMA 2020) and its extensions, to raise our concerns about this checklist.

The PRISMA Executive has established processes to guide decisions about the need for new extensions, ensure that the reporting guidelines are developed using rigorous consensus-based methods, and determine whether they ultimately should be endorsed. This is important because use of the PRISMA name not only signals that a checklist is relevant to systematic reviews but also conveys to users that the guideline has been developed using appropriate and rigorous methods. As the developers of the PRISMA-trAIce checklist did not follow our processes, we do not endorse it as a PRISMA checklist.

We would also like to take this opportunity to highlight the process for developing PRISMA extensions for the benefit of those interested in developing future extensions. This process, outlined on the PRISMA website [2], aims to ensure that PRIMSA extensions are complementary with one another and the main statement, not redundant, and are developed using consensus-based methods. Applicants are expected to complete a form providing details of their proposed PRISMA extension, which the PRISMA Executive assesses against the criteria for approving new extensions [3].

The importance of these processes is illustrated by several concerns regarding the development and positioning of PRISMA-trAIce. There is very limited information describing how the checklist items were selected for inclusion. More broadly, the reporting guideline development process does not follow established guidance for developing reporting guidelines, including extensions [4]. The authors of PRISMA-trAIce acknowledge this, stating that their work is “the result of a systematic adaptation, not a formal, large-scale consensus-building exercise.” They also describe the checklist as a foundational tool “paving the way for a formal, community-endorsed standard.” This raises the question of why the PRISMA name is being used at this stage. Inappropriately badging the checklist as a PRISMA extension could be confusing for authors, who may be unsure how to use PRISMA-trAIce alongside PRISMA 2020 and other extensions.

We agree with Holt and colleagues [1] that PRISMA 2020 should be updated to incorporate guidance on the use of AI tools in the systematic review process. To meet this need, members of the PRISMA Executive and its global collaborators are spearheading the development of such consensus-based guidance. This guidance will soon serve as an official resource, hosted on the PRISMA website to ensure findability and drive uptake across the evidence synthesis community.

## Abbreviations

PRISMA: Preferred Reporting Items for Systematic reviews and Meta-Analyses
